# Polyphenol Compounds and Biological Activity of Caper (*Capparis spinosa* L.) Flowers Buds

**DOI:** 10.3390/plants8120539

**Published:** 2019-11-25

**Authors:** Aneta Wojdyło, Paulina Nowicka, Mar Grimalt, Pilar Legua, Maria Soledad Almansa, Asunción Amorós, Ángel Antonio Carbonell-Barrachina, Francisca Hernández

**Affiliations:** 1 Department of Fruit and Vegetable Processing, Wrocław University of Environmental and Life Sciences, 37 Chełmońskiego Street, 51-630 Wroclaw, Poland; paulina.nowicka@upwr.edu.pl; 2 Department of Applied Biology, Universidad Miguel Hernández de Elche, Carretera de Beniel, km 3.2, 03312 Orihuela, Alicante, Spain; margrimalt92@gmail.com (M.G.); ms.almansa@umh.es (M.S.A.); aamoros@umh.es (A.A.); 3 Department of Plant Science and Microbiology, Universidad Miguel Hernández de Elche, Carretera de Beniel, km 3.2, 03312 Orihuela, Alicante, Spain; p.legua@umh.es (P.L.); francisca.hernandez@umh.es (F.H.); 4 Department of Agrofood Technology, Food Quality and Safety Research Group, Universidad Miguel Hernández de Elche, Carretera de Beniel, km 3.2, E-03312 Orihuela, Alicante, Spain; angel.carbonell@umh.es

**Keywords:** caper, nonpareilles, surfines, capucines, capotes, fines, gruesas, LC-qTOF-MS/MS, antioxidant activity, anti-diabetic activity, cholinesterase’s inhibition

## Abstract

The aim of the study was to analyze potential health-promoting components of caper flower buds (*Capparis spinosa* L.) at six stages of development in two cultivars. Polyphenol compounds (flavonols, hydroxycinnamic acids, flavan-3-ols) were identified by Liquid Chromatography– quadrupole Time–of–Flight –Mass Spectrofotometer/Mass Spectrofotometer (LC-qTOF-MS/MS) and quantified by Ultra Performance Liquid Chromatography–Photodiode Array-Fluorescence Detector (UPLC-PDA-FL). Moreover, antioxidant properties (ABTS+•, FRAP, and ORAC), anti-diabetic potential (α-amylase and α-glucosidase), and anti-aging activity (acetylcholinesterase (AChE) and butyrylcholinesterase (BuChE)) of the buds were examined. Total phenolic compounds in the investigated caper varied from 10,720 to 3256 mg/100 g dry weight (DW), and depended on a genotype and growing stage of caper flowers. Among six different growing stages, the one named ‘nonpareilles’ was characterized by significantly higher content of polyphenols than the remaining five stages. The flavonols in caper flowers represented a mixture of different glycosylated quercetin, kaempferol, myricetin, and isorhamnetin derivatives, accounting for 38%–67%, 15%–36%, 4%–7%, and 0.8%–3%, respectively, of total flavonols,. Their contents strongly depended on the growth stage. ‘Nonpareilles’ and ‘surfines’ were richer in flavonols than ‘fines’ and ‘gruesas’. Of the six investigated growth stages, ‘nonpareilles’ accumulated the greatest amounts of bioactive compounds that correlated with antioxidant and anti-diabetic properties, and were more potent BuChE than AChE inhibitors.

## 1. Introduction

Polyphenol compounds are secondary metabolites commonly found in different plant organs, such as in flowers, berries, fruits, roots, leaves, and stems. Currently, polyphenol compounds are receiving considerable attention for their health benefits and capability of preventing the diseases of modern civilization such as cardiovascular or diabetic (hypertriglyceridemia and hyperglycemia) disorders. They also show anti-obesity, anti-inflammatory, anti-bacterial, anti-tumor, and anti-hepatotoxic activities [1,2]. Plants have become increasingly important, not only in the pharmaceutical but also in the food industry, due to the presence of physiologically active phytochemicals capable of imparting diverse health benefits with diet [3].

Capparis species are grown for their medicinal properties and as food sources. Medicinal plants *Capparis* sp. belong to the *Capparidaceae* family. The most popular species include *C. spinosa, C. decidua,* and *C. ovata*, and less known are *C. sepiaria, C. tomentosa,* and *C. shumilis* [2,3,4,5].

Caper grows abundantly in wild arid regions of Asia, Africa, Saudi Arabia, and Europe, especially in the Mediterranean basin. Caper consumables are mainly flower buds and berries. Additionally, different parts of caper (roots) have been used in traditional Chinese, Iranian, Moroccan, Pakistani, Egyptian, and Arabian medicine [5]. Nowadays, it is widely cultivated in Mediterranean countries such as Turkey, Morocco, Algeria, as well as France, Spain, Greece, and Italy. Average annual global production of caper is estimated at 10,000 tons, and Spain is one of the main European producers, with a cultivation area of about 2,600 ha and annual production 500–1000 tons [2,4]. Capers are consumed for their flavor and digestive properties in fresh salads, pizza, and after processing as pickle or products of lactic acid fermentation [6,7].

Caper berries contain a wide range of bioactive compounds such as alkaloids, flavonoids, steroids, terpenoids, and tocopherols [2,3,4,5,7]. A few papers [2,3,4,5] have reviewed some of the chemical compounds and health benefits of *C. spinosa* in different aspects, including its potential for sustainability. However, the literature contains limited data on flavonoid identification, indicating that capers contain only quercetin, isorhamnetin, and kaempferol of -3-*O*-rutinoside [4,8,9]. NMR techniques yielded one quercetin 3*-O*-6’-α-L-rhamnosyl-6’-*O*-ß-D-glucosyl-ß-D-glucoside in *C. spinosa* berries. Other works reported the presence of some derivatives of (+)-catechin and (epi)-catechin [7] or some derivatives of benzoic acids [10].

Despite this, scientific literature on caper biological activity and polyphenols is still insufficient, especially regarding caper flowers. Health benefits, especially antioxidant potential of food, depend on the type and amount of flavonoids. Research reports published to date have compared bioactive compounds in different caper organs (flowers, berries, leaves, seeds) [11,12,13], different cultivars, and genotypes (both cultivated and wild) [14]. Some papers also investigated the influence of selected processes on the retention of bioactive compounds in final commercial or non-commercial products [6,7,15,16]. To the best of our knowledge, no paper has been published on the content of phenolic compounds in caper flowers at different stages of development, that is, ‘nonpareilles’, ‘surfines’, ‘capucines’, ‘capotes’, ‘fines’, and ‘gruesas’.

Considering the wide interest in caper, we designed this study with the aim (i) to identify and quantify polyphenolic compounds (flavonoids and hydroxycinnamic acids) from two of the most often cultivated cultivars in Spain, and (ii) to determine biological activity (antioxidant, anti-diabetic, and cholinesterase inhibition properties) at different growth stages of caper flowers: ‘nonpareilles’, ‘surfines’, ‘capucines’, ‘capotes’, ‘fines’, and ‘gruesas’.

## 2. Results and Discussion

### 2.1. Identification of Phenolic Profile

**Figure 1:**
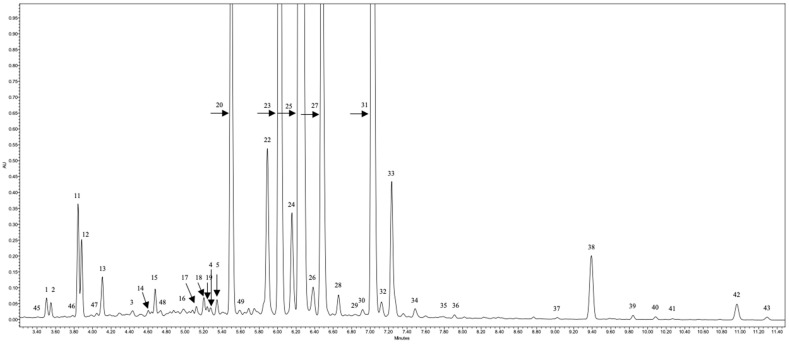
Typically chromatogram of caper flowers. The names of the compounds are presented in Table 1.

Table 1 and Figure 1 present details of Liquid Chromatography– quadrupole Time–of–Flight –Mass Spectrophotometer/Mass Spectrophotometer (LC-qTOF-MS/MS) analysis—retention times (recorded on total ion current (TIC) chromatograms), λ_max_, main ions and formulas for deprotonated molecules [M − H]^−^ and main fragment ions in MS/MS of 34 flavonols, 10 hydroxycinnamic acids, and five flavan-3-ols.

#### 2.1.1. Flavonol Glycosides

Caper flowers contained a total of 34 different flavonol compounds, including derivatives of aglycones of quercetin (*m/z* 301), kaempferol (*m/z* 285), isorhamnetin (*m/z* 315), and myricetin (*m/z* 317). An analysis of chemical structure of individual aglycones ([Aglc-H]^−^) showed a cut off for sugars at *m/z* 162 (glucose or galactose), *m/z* 146 (rhamnoside), and *m/z* 308 (rhamnohexosyl as rutinoside). We did not observe any sugars substituted by *p*-coumarate, malonate acetate, or other compounds. Some UV spectra of compounds **12**, **13**, **14**, **22**, **23**, **34**, **37**, and **42** were undetectable due to their low abundance, especially of flavonoid-*O*-tri- or –tetra-glycosides. Therefore, their identification was based on an exhibited deprotonated molecular ion of a flavonoid and/or literature data.

We identified two caper aglycones as kaempferol (**43**; *m/z* 285.95) and quercetin (**35**; Rt:11.71 min; UV 256, 268sh, 296sh, 374 nm: *m/z* 301.04). They were also detected in capers from Sardinia by Maldini et al. [14].

Compounds **35**, **38**, **40**, and **42,** belonging to flavonoid-*O*-monoglycosides, showed in their MS fragmentation a loss of 162 amu (hexosyl radical) or 146 amu (rhamnosyl radical) that yielded an ion of a deprotonated aglycone as a base peak.

In their MS/MS fragmentation pattern, a loss of 162 amu was observed for only two compounds (**35** and **38**), and the deprotonated aglycone lost either 317 amu (isorhamnetin) or 315 amu (myricetin). Therefore, the compounds were identified as isorhamnetin-3-*O*-hexoside (**35**) and myricetin-3-*O*-hexoside (**38**). Compounds **40** and **42** were characterized as mono-rhamnoside isomers of myricetin. Monoglycosides such as quercetin and isorhamnetin of -3-*O*-glucoside were previously documented by Siracusa et al. [17].

Compounds **18, 20, 21, 25, 28, 30, 31, 33**, **34**, **36**, **39**, and **41,** belonging to flavonoid-*O*-diglycosides, showed in their MS fragmentation a loss of 308 amu ((rhamno)hexosyl radical) that yielded the ion of an aglycone. Compounds **20** and **21** were characterized as myricetin-3-*O*-derivatives; compounds **18**, **31,** and **34** as isorhamnetin-3-*O*-derivatives; compounds **30**, **33**, and **36** as kaempferol-3-*O*-derivatives; and compounds **25**, **28**, **29**, **39,** and **41** as quercetin-3-*O*-derivatives. Compounds **18**, **20**, **21**, **25**, **28**, **30**, **31**, **33**, **34,** and **36** could correspond to flavonol-3-*O*-rutinoside ((rhamno)hexosyl radical—308 amu). In the MS fragmentation of compounds **39** and **41,** the losses of 146 amu (rhamnosyl radical) and 162 amu (hexosyl radical) indicated that these two sugars were situated on different phenolic hydroxyl groups of aglycones. However, Abu-Reidah, Gil-Izquierdo, Medina, and Ferreres [18] and Ferreres, Grosso, Gil-Izquierdo, Fernandes, Valentão, and Andrade [19] suggested a presence of an interglycosidic 1→2 bond. Additionally, the loss of 146 + 18 amu demonstrated that these compounds usually have the 1→2 bond.

Moreover, a loss of two sugars as 324 amu (di-hexosyl radical) from compound 29 indicated the presence of diglycosides with an interglycosidic 1→6 bond, which is very difficult to break down, as suggested by numerous authors [18,20]. Therefore, this compound can be characterized as quercetin-3-*O*-hexoside-hexoside (**29**). As already indicated, similar results were reported by other authors [19]. The flavonoid-*O*-diglycoside group contained some pairs of compounds, for example, quercetin-3-*O*-rutinoside (**25/28**), kaempferol-3-*O*-rutinoside (**33/36**)**,** isorhamnetin-3-*O*-rutinoside (**31/34**), and myricetin-3-*O*-rutinoside (**20/21**). All the pairs are a combination of structure or confirmation type of sugar (i.e., glucose, galactose).

Some diglycosideflavonols, for example, quercetin-, kaempferol-, and isorhamnetin-3-*O*-rutinoside were previously identified in fermented caper berries [7] as documented by Siracusa et al. [17]. However, Inocencio et al. [8] found only quercetin- and kaempferol-3-*O*-rutinoside in different commercial pickled capers produced in Mediterranean countries.

The second largest group of chemicals with a characteristic structure of flavonoid-*O*-triglycosides identified in capers included compounds **11**, **15**, **16, 17, 19**, **22**–**24**, **26, 27**, **32**, and **37**. The compounds classified as flavonoid-triglycosides that after deprotonation yielded the ion of the aglycone as base peak turned out to be quercetin (**11**, **15**, **16**, **22**, **24**), kaempferol (**17**, **26**, **32**, **37**), and isorhamnetin (**19**, **23**, **27**).

MS/MS fragmentation of the resulting ions [((M − H)-162)]- (**11**, **15**, **16**, **17**, **19**, **23**, **32**), derived from some triglycoside compounds, was caused by a separation of a hexoside from the rest of the molecule (aglycon + 308 amu). The glycosidic fraction in position 3, indicating diglycosides with an interglycosidic 1→6 bond, was very difficult to break down. Additionally, in these types of compounds, we identified some pairs (**15**/**16**, **17**/**32,** and **19**/**23**) labeled as quercetin-, kaempferol-, and isorhamnetin of -3-*O*-rutinoside-7-hexoside, respectively. 

Losses of [(M − H) -146 (rhamnosyl radical)] in compounds **22, 24,** and **37** yielded the deprotonated aglycone + 308 amu (rhamnoside). These compounds were identified as derivatives of -3-*O*-rutinoside-7-*O*-rhamnoside of quercetin (**22**/**24**) and kaempferol (**37**). Similar compounds were identified in edible parts and by-products of date palm (*Phoenix dactylifera* L.) [18].

Compounds **26** and **27** presented deprotonated molecular ions other than the remaining flavonoid-*O*-triglycosides (Table 1). After a loss of one hexose and two rhamnoses, their MS spectra showed a deprotonated aglycone of kaempferol (*m/z* 285). Additionally, the loss of 266 amu (146 + 120) fragment was observed in the MS of a compound produced by the internal cleavage of a rhamnose (−146 amu) at position 6 and involved a hexose from positions 0 and 2 (−120 amu). Similarly to other researchers [20], we found that the loss of 164 (146 + 18, radical rhamnosyl + water) and 146 (radical rhamnosyl) indicated a new glycosylation with another rhamnose moiety. These compounds were then identified as -3-*O*-(2-rhamnoside)-rutinoside of kaempferol and isorhamnetin, respectively. Compound **26** was previously identified in fermented caper berries [7].

Compounds **12**, **13,** and **14** with the highest molar mass at *m/z* 917.11, 901.12, and 931.08 were quercetin-, kaempferol-, and isorhamnetin of -3-*O*-rutinoside-hexoside-7-*O*-rhamnoside, respectively. MS/MS fragmentation showed ions obtained after losing the rhamnosyl at position 7 ([M − H-146). The hexoside must be localized at position 6, as inferred by the presence of the ion [(M − H-162) produced by an internal cleavage of the hexose at positions 0 and 2 and involving the rutinoside at position 6, which is of difficult fragmentation. As far as we know, flavonoids with these masses have not been identified before in capers, but were detected in other plants such as *Bauhinia forficate* L. by Ferreres et al. [16].

Some compounds identified in this work, for example, quercetin-3-*O*-rutinoside, quercetin-3-*O*-glucoside, quercetin-3-*O*-glucoside-7-*O*-rhamnoside, kaempferol-3-*O*-rutinoside, kaempferol-3-*O*-rhamnorutinoside, and isorhamnetin-3-*O*-rutinoside were also identified in wild caper berries [17,21,22,23]. Kaempferol-3-rutinoside, quercetin-3-rutinoside, quercetin-7-rutinoside, and quercetin-3-glucoside-7-rhamnoside were isolated from stems and leaves of *C. spinosa* [22]. *C. spinosa* also produced quercetin-3-*O*-[6′-rhamnosyl-6′-glucosyl]-glucoside [22]. Apart from these compounds, compounds **12**–**14, 17, 19, 23, 24, 26,** and **27** have never before been identified and quantified in caper berries.

#### 2.1.2. Flavan-3-ols

Our caper flower samples yielded five compounds in monomer, dimer, and trimer form, belonging to flavan-3-ols. Identification of compounds **45**, **46**, **47**, and **48** was attained by their comparison with authentic standards. Their *m/z* 289 was exactly the same as for commercial compounds, allowing us to identify compounds **45** and **46** as (+)-catechin and (−)-epicatechin, respectively. Compounds **47** and **49** are a dimer and a trimer of procyanidins with characteristic *m/z* 577 and 865, respectively. MS/MS spectra of these ions showed the retro-Diels−Alder fragmentation as a loss of phloroglucinol A-ring (loss of 126 amu), of heterocycles (loss of 152 amu), and rupture of the interflavan linkage (loss of 288 amu). The last polymeric procyanidin was characterized as procyanidin dimer (**49**) with *m/z* 577, and after MS/MS with *m/z* 289 and 245. Previously, only Jiménez-López et al. [7] identified two dimers and two trimers of procyanidins and (−)-epicatechin.

#### 2.1.3. Hydroxycinnamic Acid Derivatives

Hydroxycinnamic acid derivatives were the second group after flavonol glycosides that contributed to the final concentration of polyphenols in caper flowers. Compounds **1**–**10** were identified on the basis of their mass and UV spectra characteristic of hydroxycinnamic acid derivatives. Only a few hydroxybenzoic acids (cinnamic acid, *p*-hydroxybenzoic acid, protocatechuric acid, and vanillic acid) and only a few hydroxycinnamic acids (chlorogenic acid, ferulic acid, coumaric-glucoside, 4-feruloylquinic acid, and sinapic acid) were previously identified [24], but in different works and in different parts of *C. spinosa*, such as stem, leaves, flowers, roots, or berries [7,14,17]. Two compounds (**1** and **9**) had fragmentation typical of *p*-coumaric acid at *m/z* 163 and 119 after decarboxylation of coumaric acid ([M − H]^−^ -CO_2_). Compound **9** showed an ion at *m/z* 325 with a daughter ion *m/z* 163 ([M − H)^−^ -hexose), most probably glucose. Therefore, compounds **1** and **9** were tentatively annotated as *p*-coumaric and coumaric acid-*O*-hexoside, respectively. The presence of coumaric acid-*O*-hexoside was previously reported by other authors [7].

Compounds **2** and **3** exhibited a deprotonated molecular ion at *m/z* 353, and also yielded a fragment ion at *m/z* 191. The MS/MS spectrum *m/z* 191 was likely due to quinic acid ([quinic acid-H]^−^) ion resulting from a cleavage of the C–O bond of the ester linkage typical of caffeoylquinic acid. Compound **3** yielded near equal *m/z* 173 and 179 at MS/MS, whereas for compound **2,** *m/z* 179 predominated. Therefore, compounds **2** and **3** were identified as 5-caffeoylquinic acid and 4-caffeoylquinic acid, respectively. They were also compared with authentic standards. The present identification corroborated the data published for caper berries by Maldini et al. [14] and Siracusa et al. [17].

Mass spectra of compounds **4** and **5** revealed dominant ions at *m/z* 337, and gave anion signals at *m/z* 191 (quinate) and low intensity ion *m/z* 163 (coumarate), characteristic of 5-*p*-coumaroylquinic acid. Targeted MS/MS experiments showed the same fragmentation patterns without any other intense ions [24]. This confirmed that compounds **4** and **5** were *trans*-5-*p*- and *cis*-5-*p*-coumaroylquinic acid, respectively. 5-*p*-Coumaroylquinic acid was the most abundant *p*-coumaroylquinic acid and was previously identified in caper berries by Siracusa et al. [17].

Fragment ions after MS/MS at *m/z* 191 ([M − H]^−^—quinic acid) and 173 ([M − H]^−^—ferulic acid) were observed in three compounds (**6**–**8**), indicating them as derivatives of quinic and ferulic acids with a characteristic pseudomolecular ion at *m/z* 367.

These three compounds are feruloylquinic acid (FQA) with region-isomers eluted as 3-, 5-, and 4-feruloylquinic acid. The three positional isomers were identified by their distinct fragmentation—3-FQA gave an intense MS/MS ion at *m/z* 193 [ferulate], whereas 4-FQA yielded an abundant *m/z* 173 ion and weak ions *m/z* 193 and 191, and for 5-FQA we measured a strong ion at *m/z* 191 (quinate) and a weak ion at *m/z* 173, as suggested by Parveen et al. [24] and Jaiswal et al. [25]. An earlier paper [14] confirmed only the presence of ferulic acid in caper berries, and other authors identified one of these isomers (4-FQA) [17].

We found *m/z* 223.06 with fragments *m/z* 205 ([M − H]^−^—H_2_O), 179 ([M − H]^−^—CO_2_), and 163 ([M − H]^−^—OCH_3_-OCH_3_). This compound was evaluated as sinapic acid (**10**) after comparison with an authentic reference substance. Sinapic acid is widely distributed in the Brassicaceae family, and Capparis belongs to Capparidaceae, a family closely related to Brassicaceae. The presence of sinapic acid has already been reported in caper leaves [26].

### 2.2. Phenolic Compounds’ Quantitative Profile

Phenolic composition of capers at different growing stages has not been recognized so far. Total phenolic compounds in the investigated caper varied from 10720 to 3256 mg/100 g dry weight (DW) (Table 2), and depended on a genotype and growing stage.

Phenolic content in Orihuela n^o^ 7 (ORI.7) sample was significantly higher than in Orihuela n^o^ 10 (ORI.10). Among six different growing stages, the one called ‘nonpareilles’ was characterized by significantly higher content of polyphenols than the other stages. Nonpareilles caper flowers of ORI.7 showed three times higher content of phenolic compounds than ‘fines’ or ‘gruesas’ flowers, but for ORI.10 the differences were smaller. The content of phenolic compounds in caper is higher than in some exotic and common fruits and vegetables. Consumers value food with a high level of bioactive substances, such as polyphenols, as they are known to be the most abundant antioxidants in our diet.

Our analyses of caper phenolic compounds identified flavonols as the major polyphenolic group, representing on average 80% to 95% of all phenolics, irrespective of the growing stage. Flavan-3-ols (with abundance of 3% to 14%) took the second place, and phenolic acids (1% to 5%) were the third. These results corroborated those from previous reports [6,9,15], but our work focused mainly on flavonol content. High content of flavonols may reflect plant response to biotic and abiotic stress or acclimation to environmental stressors such as heat, cold, UV radiation, drought, salinity, or an attack of herbivores or pathogens [27]. Several studies showed the effect of temperature and sunlight on flavonoid accumulation in the flower and berry or fruit skin. Wang and Zheng [27] found that strawberries grown at 18/12 °C generally had the lowest anthocyanin, flavonol (quercetin-3-*O*-glucoside and quercetin-3-*O*-glucoronide), and phenolic acid contents, whereas at 30/22 °C their content was the highest. Solovchenko and Schmitz-Eiberger [28] demonstrated quercetin glycosides to be the principal group of flavonoids accumulated in apple skin in response to high sunlight. Lee et al. [29] reported a positive correlation between the duration of exposure to sunshine and flavonoid content in the leaves of *Angelica keiskei*.

Ultraviolet C (UV-C) induced accumulation of flavonols and activity of the enzymes of phenylpropanoid pathway at the beginning of growth during the first 3 days, but later on the effects ceased [30]. Additionally, strong sun exposure improves synthesis and glycosylation of flavonols with various sugar molecules and synthesis of larger number of compounds from the flavonoid biosynthesis pathway, with dihydrokaempferol serving as a parent compound for kaempferol, quercetin, isorhamnetin, and myricetin [30].

These results confirm that capers are a very rich source of phenolic compounds, especially flavonols, flavan-3-ols, and hydroxycinnamic acid. Total flavonols in caper flowers comprise a mixture of different glycosylated quercetin, isorhamnetin, kaempferol, and myricetin derivatives.

Quercetin, kaempferol, myricetin, and isorhamnetin derivatives represented, respectively, 38%–67%, 15%–36%, 4%–7%, and 0.85–3% of total flavonols in caper flowers, but their contents strongly depended on the growth stage. ‘Nonpareilles’ and ‘surfines’ were richer in flavonols than fines and gruesas. The content of flavonols in ‘gruesas’, as compared with ‘nonpareilles’ dropped by 4.3, 1.5, 5.2, and 5.2 times for quercetin, kaempferol, isorhamnetin, and myricetin derivatives in ORI.7 capers. A similar tendency was observed for ORI.10 capers. The smallest differences between individual stages were spotted for ‘nonpareilles’ and ‘surfines’ or for ‘capucines’ and ‘capotes’. Small caper flowers of the size below 7–8 mm were richer in flavonols than larger flowers. A similar effect was observed for honeysuckle berries and the content of anthocyanins [31].

Diglycoside structure (77%–85%) of flavonols predominated over monoglycosides (11%–21%), triglycosides (2%–6%), and tetraglycosides or aglycone (<2%). Quercetin-3-*O*-rutinoside and kaempferol-3-*O*-rutinoside were the major flavonols. The most abundant among the remaining identified flavonoids were myricetin-3-*O*-rutinoside, isorhamnetin-3-*O*-rutinoside-7-*O*-hexoside, and isorhamnetin-3-*O*-hexoside (Table 2). Siracusa et al. [17] and Inocencio et al. [8] reported -3-*O*-rutinoside of quercetin, kaempferol, and isorhamnetin as major flavonoids in *C. spinosa*, which was concurrent with this work.

The minor flavonols in caper flowers identified for the first time included isorhamnetin-3-*O*-rutinoside-hexoside-7-*O*-rhamnoside, kaempferol-3-*O*-rutinoside-7-*O*-hexoside, myricetin-3-*O*-hexoside, isorhamnetin-3-*O*-rutinoside-7-*O*-hexoside, and isorhamnetin-3-*O*-(2-rhamnoside)-rutinoside.

Quercetin-3-*O*-rutinoside content in caper flowers was higher than in onion (~120 mg/100 g), thyme (~ 2490 mg/100 g), or buckwheat (~5350 mg/100 g), and it was one of the most common quercetin glycosides [8]. Caper flowers therefore seem to be an abundant source of quercetin-3-*O*-rutinoside and the other flavonols. Inocencio et al. [8] suggested that 10 g of *C. spinosa* would provide approximately 65 mg of flavonoid glycosides in our diet. Flavonol derivatives, especially quercetin, are very important for human health [18]. Their consumption reduces the risk of cardiovascular disease due to their anti-hypertensive and anti-platelet aggregating properties, and decreases low-density lipoprotein (LDL) cholesterol levels [17]. Flavonol accumulation in fruit skin as a result of sunlight exposition is well documented and is the most important environmental factor inducing flavonol biosynthesis. Fruits with sun-exposed peel have higher levels of anthocyanins and flavonols than those grown in the shade [30].

Polymeric procyanidins classified as flavan-3-ols were determined using phloroglucinol methods. They showed that polymeric procyanidins in caper flowers consisted mainly of polymer unit of (−)-epicatechin rather than (+)-catechin. In our study, polymeric procyanidins in the total phenolic pool accounted for no more than 14%, and their concentration ranged from 200 mg to 700 mg/100 g. Jiménez-López et al. [7] postulated that fermented caper still contained (–)-epicatechin and (epi)catechin dimer and trimer in the amount of 160 mg/100 g. Regarding flavonols, ’nonpareilles’ had similar content of polymeric procyanidins to ‘gruesas’. ‘Capucines’, ‘capotes’, and ‘fines; exhibited higher levels of polymeric procyanidin than capers at other growth stages. Zhang et al. [1] reported that accumulation of flavan-3-ols in Cabernet Sauvignon grape depended on a developmental stage and corresponded to supplemental UV light. In fact, UV radiation increased the levels of flavan-3-ol during the berry development but not in the mature berries [1]. Francesca et al. [6] and Maldini et al. [14] did not identify any compounds belonging to flavan-3-ols in caper berries.

Quantitative analysis of polymeric procyanidins revealed their higher content in ORI.7 capers versus ORI.10 sample. Some researchers [8] reported that flavan-3-ols present in caper extracts might be responsible for antiparasitic activity and act as synthetic phenolic anthelmintics.

Hydroxycinnamic acids belonged to a minor group of caper flower polyphenols. Their concentration ranged from 178.6 to 71.8 mg/100 g for ORI.7 and from 283.4 to 44.8 mg/100 g for ORI.10. Similarly to flavonols, the content of hydroxycinnamic acids in ‘nonpareilles’ was higher (1.2 and 6.3 times for ORI.7 and ORI.10, respectively) than in ‘gruesas’. The predominant hydroxycinnamic acids in caper flowers were feruloylquinic acid (19%–51% and 45%–61% for ORI.7 and ORI.10, respectively) and caffeoylquinic acid (17%–32% and 5%–26% for ORI.7 and ORI.10, respectively). The remaining acids were present at low concentrations, that is, sinapic acid constituted less than 2%–14%. Literature data on hydroxycinnamic acids are sparse.

Siracusa et al. [17] identified 5- and 4-caffeoylquinic, 5-*p*-coumaroylquinic, and 4-feruloylquinic acids, and postulated 5-caffeoylquinic and 4-feruloylquinic acids as the major compounds. Jiménez-López et al. [7] identified some hydroxycinnamic acids in fermented caper (coumaric acid-*O*-hexoside), but did not quantify them.

Hydroxycinnamic acids such as 5-caffeoylquinic and caffeoylquinic acid are good sources of antioxidants *in vitro* that protect low-density lipoprotein (LDL) from oxidation and, therefore, supposedly prevent various age-related diseases. Sinapic acid present in caper flowers conveys their bitter taste and astringency, similarly to rape products.

### 2.3. Biological Potential of Caper Flowers

We evaluated biological potential of caper flowers at different growth stages on the basis of their antioxidant activity (2,2′-azinobis-(3-ethylbenzothiazoline-6-sulfonic acid)] radical cation (ABTS^•+^), ferric-reducing antioxidant power (FRAP), and Oxygen Radical Absorbance Capacity (ORAC), antidiabetic activity (α-amylase and α-glucosidase), and cholinesterase inhibition (acetylcholinesterase (AChE) and butyrylcholinesterase (BuChE)). The biological potential of caper flowers clearly depended on their content of bioactive compounds and growth stage.

The ORAC for nonpareilles was significantly higher than for ‘gruesas’ (1.7 times for ORI.7 and 1.9 times for ORI.10). ‘Nonpareilles’ capers of ORI.7 exhibited higher antioxidant activity (27.7 mM Trolox/100 g) than ‘nonpareilles’ ORI.10 (19.3 mM Trolox/100 g) (Table 3). Capers at ‘gruesas’ stage had the lowest antioxidant activity of 16.8 and 10.7 mM Trolox/100 g for ORI.7 and ORI.10, respectively. The ABTS and FRAP assays showed the same trend as the ORAC assays.

α-Amylase and α-glucosidase catalyze digestion of oligo- and disaccharides into absorbable monosaccharides. Caper flower extracts inhibited these enzymes, with IC_50_ values ranging from 3.74 to 0.93 mg/mL for α-glucosidase and from 3.68 to 1.52 mg/mL for α-amylase. ORI.7 ‘nonpareilles’ extract turned out to be an effective potential inhibitor of α-glucosidase and α-amylase. This was not true for the ORI.10 sample, where ‘gruesas’ extract much more effectively inhibited α-amylase and ‘capotes’ extract showed high inhibitory potential towards α-glucosidase. Our results are comparable with those reported by Adriano Mollica et al. [26], where aqueous extracts of *C. spinosa* exhibited a potent anti-hyperglycemic activity in diabetic rats. Other authors [32] suggested alkaloids, saponins, terpenes, and phenolics as major compounds responsible for anti-diabetic effect.

As far as we know, this is the first report on cholinesterase inhibition by caper flowers. The ability of caper flowers to inhibit AChE and BuChE was evaluated *in vitro*. In all cases, the inhibition closely depended on the growth stage and cultivar. In the ORI.7 sample, maximum inhibition of AChE and BuChE reached 18.3% and 31.0%, respectively, and in ORI.10 sample it was 28.1% and 33.8%, respectively. ‘Nonpareilles’ flowers turned out to be the most potent inhibitors. Moreover, the suppression of BuChE was more effective than that of AChE. This is desirable, as peptidase activity of butyrylcholinesterase controls the development and progression of Alzheimer’s disease. The extracts capable of BuChE inhibition may also prevent the disease progression caused by β-amyloid protein accumulation, as they help to diffuse the β-amyloid plaques [33]. Alzheimer’s disease is the most common cause of dementia in the elderly, being characterized by degeneration of cholinergic neurons in specific areas of the brain associated with higher intellectual functions, memory, and consciousness [16].

Polyphenolics are known for their high biological activity that is determined by their individual composition. Ferreres et al. [16] postulated that antioxidant activity of flavonoids results from their structure, especially a free hydroxyl group at 4’ present in all derivatives, a double bond between C2-C3 present in all identified compounds, a free hydroxyl group at 3′ found only in quercetin and kaempferol (**34**, **35**; Table 1), and a di-hydroxyl group at *orto* position in B-ring found only in quercetin and myricetin derivatives. Therefore, a possible explanation of high antioxidant activity of caper flowers, especially at ‘nonpareilles’ stage, is high concentration of quercetin and its derivatives.

Studies focused on anti-diabetic properties and cholinesterase inhibition often investigated the structure of analyzed compounds. Mandatory structural features for anti-AChE activity include the presence of *O*-glycosylation at C7 rather than at C3 that diminishes cholinesterase inhibition, a double C2–C3 bond present only in some compounds identified in caper flowers, and methoxylation at C4, not detected in caper flowers but determined for C3 in isorhamnetin derivatives that had no effect on this activity [19,34,35]. For BuChE inhibition, methoxylation at C4 is not so important [36]. Quercetin-3-*O*-galactoside (absent in caper flowers) at 90 µg/mL was capable of reducing AChE by 55% [36]. Caper flowers contained mainly compounds with -3-*O*-glycosylation (mono, di, tri, tetra), so we did not expect them to be effective cholinesterase inhibitors.

As postulated by Ferreres et al. [16], anti-diabetic activity requires a hydroxyl group at position 4′ typical for quercetin, myricetin, and kaempferol structure, and a hydroxyl group at position 3′ typical for compounds of quercetin, isorhamnetin, and myricetin structure.

Table 4 presents Pearson’s correlation coefficient for polyphenol content and biological activity: antioxidant activity (ORAC, ABTS, FRAP), cholinesterase inhibition (AChE, BuChE), and antidiabetic activity (α-amylase and α-glucosidase). Significant and strong correlation can be noticed between ORAC and flavonols (sum of quercetin, kaempferol, isorhamnetin, and myricetin derivatives; above *r* = 0.684) and phenolic acid (sum of caffeic acid, *p*-coumaroylquinic acid, and feruloylquinic acid). We also found a positive correlation between phenolic compounds and cholinesterase inhibition (AChE, BuChE) and antidiabetic activity (α-amylase and α-glucosidase).

However, we only noticed a negative correlation between polymeric procyanidins and the biological activity test, except for BuChE, where it was not significant.

Mono- and di-flavonols played a greater role in biological activity (especially antioxidant activity (ORAC), α-amylase, and BuChE inhibition) than aglycone or tetra-flavonols. Additionally, phenolic acids showed higher anti-diabetic potential and anticholinesterase activity than antioxidant activity (FRAP > ABTS > ORAC). These outcomes demonstrated that we should not ignore the role of non-phenolic components of caper flowers, as they may exhibit still unknown synergic or antagonist effects. This section may be divided by subheadings. It should provide a concise and precise description of the experimental results, their interpretation, as well as the experimental conclusions that can be drawn.

## 3. Materials and Methods

### 3.1. Chemicals

Flavonols (quercetin-, kaempferol-, myricetin, and isorhamnetin: -3-*O*-glucoside, -3-*O*-galactoside, -3-*O*-rutinoside) and hydroxycinnamic acid (sinapic, *p*-coumaric, 5-caffeoylquinic acids) with a purity of HPLC standards were obtained from Extrasynthese (Lyon, France). The LC–MS and chromatography grade solvents as methanol and acetonitrile were procured from POCH S.A. (Gliwice, Poland). Formic acid and the remainder of solvents were purchased from Sigma-Aldrich (Darmstadt, Germany). Deionized water was made using a HLP SMART 1000s purification system (Hydrolab, Gdansk, Poland). The supernatant before all LC–MS and UPLC analysis was filtered through a Hydrophilic PTFE 0.20 µm membrane (Millex Samplicity Filter; Merck, Darmstadt, Germany).

### 3.2. Plant Material

**Figure 2:**
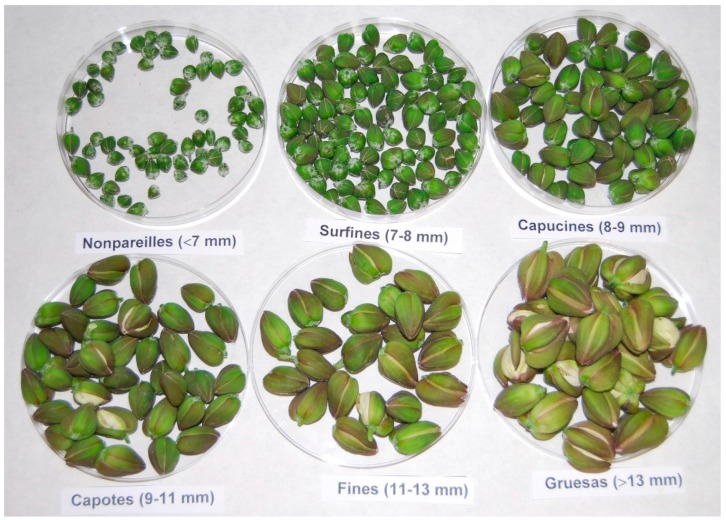
Flower bud classification by size.

The caper flowers (*Capparis spinosa* L.) at six stages of development of cultivars Orihuela n^o^7 (ORI.7) and Orihuela n^o^10 (ORI.10) were collected at the experimental field station of Miguel Hernández University in the province of Alicante, Spain (38°5’N, 0°56’W, 23.6 masl) and classified by size (Figure 2) as ‘nonpareilles’ (∅ 0–7 mm), ‘surfines’ (∅ 7–8 mm), ‘capucines’ (∅ 8–9 mm), ‘capotes’ (∅ 9–11 mm), ‘fines’ (∅ 11–13 mm), and ‘gruesas’ (∅ > 13 mm) (Boletin Oficial del Estado, 1984). The samples were hand-collected provided from University Miguel Hernández, Orihuela, Spain, in September 2017, and were taxonomically identified by an expert botanist from the Department of Plant Sciences and Microbiology using the protocol by García-Rollán [37]. One voucher of each cultivar is kept in the Miguel Hernández University herbarium (#072010 and #102010). All samples were freeze-dried, ground in a laboratory mill, and stored at −80 °C to prevent degradation until analysis.

### 3.3. Separation, Identification, and Quantification of Polyphenolic Compounds

For polyphenolic compounds, three individual sample replicates from each freeze-dried caper (max. 0.5 g each) were extracted by 5 mL of 1% formic acid in a 30% methanol solution, as described previously by Wojdyło et al. [38]. Identification and quantification of phenolic compounds by LC-qTOF-MS/MS and UPLC-PDA-FL, respectively, was analyzed as described previously by [38]. The analysis of polymeric procyanidins by phloroglucinol method was performed according to the protocol described previously by [39]. The results were expressed as miligrams per 100 grams dry weight (DW).

### 3.4. Determination of Biologically Activity: Anti-Oxidant, Anti-Diabetic, and Cholinesterase Inhibition

Freeze-dried caper samples weighing (~0.5 g) were extracted with 10 mL of methanol:H_2_O:acetic acid (80%:20%:1%, v/v/v) following the procedure described previously by Wojdyło et al. [38].

The ABTS^•+^ (2,2′-azine-bis-(3-ethylene-benzothiazoline-6-sulfonic acid) scavenging test was based on measuring the decrease in the color intensity inversely proportional to the antioxidant content measured by Re et al. [39]. An ABTS^•+^ solution was prepared with an absorbance of 0.700 ± 0.02 at a wavelength of 734 nm. Caper extracts and the ABTS^•+^ solution were mixed, and after 6 min the absorption at the wavelength was measured as above. Distilled water was the blank.

The FRAP method involves determining the ability to reduce Fe3+ ions by antioxidant substances contained in sea buckthorn extracts to the blue Fe^2+^ ions complex, according Benzie et al. [40]. The absorbance of the caper extract was measured 10 min after the addition of FRAP reagent (acetate buffer, 2,4,6-Tris(2-pyridyl)-s-triazine, in HCl, and FeCl_3_ × 6H_2_O in a volume ratio of 10:1:1, v:v:v) at a wavelength of 593 nm.

The analysis of oxygen radical absorbance capacity (ORAC) consists of spectrofluorometric measurement of the decrease in fluorescence caused by oxidation of a fluorescent substance under the influence of free radicals; according Ou et al. [41]. Samples containing sea buckthorn extract, phosphate buffer, and fluorescein were incubated at 37 °C throughout the analysis period. 2,2’-Azobis(2-amidinopropane)dihydrochloride was added and spectrofluorometric measurement and was performed every 5 min at excitation wavelength 493 nm and emission wavelength 515 nm. The blank was a phosphate buffer. The antioxidant activity of the tested samples was obtained by comparing the surface under the fluorescence decrease curves over time with the surface for Trolox solution.

The antioxidant activity (ABTS^•+^, FRAP, ORAC) was expressed as mmol of Trolox per 100 grams.

The α-amylase and α-glucosidase inhibitory effect of the caper extracts was assayed according to the procedure described previously by Wojdyło et al. [38,42].

Briefly, analysis of anti*-α*-amylase inhibitory activity was based on spectrophotometric measurement of color change as a result of reaction of iodine in potassium iodide with remaining starch after enzymatic hydrolysis. After incubation at 37 °C, for caper extract samples with starch solution and *α*-amylase, the reaction was stopped using 0.4 M HCl, and solution of potassium iodide was added to obtain color. Reference samples contained phosphate buffer instead of enzyme. Absorbance was measured at 600 nm.

The analysis of *α-*glucosidase inhibitory activity consisted of the reaction of the enzyme with a β-D-glucosidase substrate, producing a yellow solution upon cleavage. Basic samples containing caper extract and enzyme were incubated as above. After addition of substrate, the mixture was incubated again at 37 °C and measurement was made at 405 nm. As in the above analysis, the reference samples contained buffer instead of enzyme.

The results of *α-*amylase and *α-*glucosidase were expressed as IC_50_ (mg/mL).

Cholinesterase inhibition was measured as acetylcholinesterase (AChE) and butyrylcholinesterase (BuChE) methods described by [38,43]. The reaction mixture was composed of caper extract sample, Tris–HCl buffer (pH 8), acetylthiocholine iodide or S-butyrylthiocholine iodide, and 5,5′-dithiobis(2-nitrobenzoic acid), and after incubation at 37 °C for 10 min, AChE or BuChE solution was added. Absorbance was measured after 15 min at a wavelength of 412 nm. The results were expressed as percent of inhibition.

All determinations of biological activity were assayed in triplicate and performed using the UV-2401 PC spectrophotometer (Shimadzu, Kyoto, Japan).

### 3.5. Statistical Analysis

Mean values ± standard deviation were conducted for polyphenolic compounds in caper samples. The mean values were subjected to analysis of variance and Duncan’s multiple range test for mean comparison (*p* = 0.05) and Pearson’s correlation by using Statistica version 13.0 (Stat-Soft, Kraków, Poland).

## 4. Conclusions

This is the first study that characterized the phenolic profile of caper flowers by LC–qTOF-MS/MS and provided a fingerprint for future quality control of this species. The research confirmed that caper flowers are a valuable source of polyphenolics with biological activity. The main constituents of the flowers included flavonols (quercetin, kaempferol, myricetin, and isorhamnetin), phenolic acids, and flavan-3-ols. Nine compounds were reported for the first time in caper flowers, which have never before been identified and quantified in caper berries. Total phenolic compounds in the investigated caper varied from 10,720 to 3256 mg/100 g DW, and depended on a genotype and growing stage. Of the six investigated growth stages, nonpareilles accumulated the greatest amounts of bioactive compounds that correlated with anti-oxidant and anti-diabetic properties, and were more potent BuChE than AChE inhibitors. Both analyzed caper cultivars (ORI.7 and ORI.10) are promising, but should be constantly improved through breeding programs to refine their functional properties. Total polyphenol compounds in nonpareilles was 3982.9 and 3693.9 mg/100 g DW for ORI.7 and ORI.10, respectively, and the dominant fraction were flavanols (~80%–85% of total polyphenol fraction). Further studies on caper flower composition should investigate nutritional values, and it is necessary to have more data from clinical trials and toxicity tests.

## Figures and Tables

**Table 1:** Phenolic compounds identified by qTOF-MS/MS in caper flowers.

No.	Name of Compounds	R_t_	λ_max_	MS [M − H]–(*m/z*)	MS/MS [M − H]−(*m/z*)			
**Hydroxycinnamic Acid**								
1	*p*-Coumaric acid	3.41	310	163.04	119.05			
2	5-Caffeoylquinic acid	3.51	285, 326	353.04	191.06/179.03/173.05			
3	4-Caffeoylquinic acid	4.43	285, 326	353.01				
4	*trans*-5-*p*-Coumaroylquinic acid	5.29	311	337.04	163.05			
5	*cis*-5-*p*-Coumaroylquinic acid	5.34	311	337.04	163.05			
6	3-Feruloylquinic acid	5.59	325	367.05	193.03/191.02/173.09			
7	5-Feruloylquinic acid	9.39	325	367.05	191.02/135.06			
8	4-Feruloylquinic acid	9.54	325	367.05	193.03/173.09/134.09			
9	Coumaric acid-*O*-hexoside	10.26	275, 314	325.03	163.11/119.12			
10	Sinapic acid	10.95	337	223.06	205.13/179.03/164.06			
**Flavonols**					−308	−162	−146	aglycone
11	Quercetin-3-*O*-rutinoside-7-hexoside	3.85	285, 352	771.06	463.06	609.06		301.02
12	Quercetin-3-*O*-rutinoside-hexoside-7-*O*-rhamnoside	3.93		917.11	609.11	755.11	463.04	30.,02
13	Kaempferol-3-*O*-rutinoside-hexoside-7-*O*-rhamnoside	4.25		901.12	593.12	739.12		285.95
14	Isorhamnetin-3-*O*-rutinoside-hexoside-7-*O*-rhamnoside	4.45		931.08		607.08	623.08	315.05
15	Quercetin-3-*O*-rutinoside-7-*O*-hexoside	4.50	202, 256, 351	771.06	463.06	609.06		301.02
16	Quercetin-3-*O*-rutinoside-7-*O*-hexoside	4.90	266, 352	771.06	463.06	609.06	625.06	301.02
17	Kaempferol-3-*O*-rutinoside-7-*O*-hexoside	5.04	342	755.07		593.07		285.95
18	Isorhamnetin-3-O-rutinoside	5.09	271, 337	623.03	315.03			315.00
19	Isorhamnetin-3-*O*-rutinoside-7-*O*-hexoside	5.21	264, 326	785.07	477.07	623.07		315.05
20	Myricetin-3-*O*-rutinoside	5.29	274, 355	625.02	317.02			317.09
21	Myricetin-3-*O*-rutinoside (isomer)	5.46	274, 355	625.03	317.03			317.09
22	Quercetin-3-*O*-rutinoside-7-*O*-rhamnoside	5.56		755.07	447.07		609.07	301.02
23	Isorhamnetin-3-O-rutinoside-7-*O*-hexoside	5.69		785.08	477.08	623.08		315.08
24	Quercetin-3-*O*-rutinoside-7-*O*-rhamnoside (isomer)	5.90	254, 353	755.07	447.07		609.07	301.02
25	Quercetin-3-*O*-rutinoside	6.33	252, 348	609.03	301.03			301.02
26	Kaempferol-3-*O*-(2-rhamnoside)-rutinoside	6.41	264, 347	739.08	431.08		593.08	285.95
27	Isorhamnetin-3-*O*-(2-rhamnoside)-rutinoside	6.50	253, 347	769.08	461.08		623.08	315.95
28	Quercetin-3-*O*-rutinoside (rutin)	6.66	256, 354	609.03	301.03			301.02
29	Quercetin-3-*O*-hexoside-7-*O*-hexoside	6.80	253, 330	625.02		463.02		301.02
30	Kaempferol-3-*O*-rutinoside	6.90	254, 346	593.03	285.03			285.95
31	Isorhamnetin-3-*O*-rutinoside	7.02	255, 266sh, 351	623.04	315.04	461.04		315.03
32	Kaempferol-3-*O*-rutionoside-7-*O*-hexoside	7.28	264, 348	755.07	447.07	593.07		285.95
33	Kaempferol-3-*O*-rutinoside (isomer)	7.38	254, 348	593.03	285.03			285.95
34	Isorhamnetin-3-*O*-rutinoside (isomer)	7.55	253, 352	623.04	315.04			315.03
35	Isorhamnetin-3-*O*-hexoside)	7.83	254, 336, 365	477.04		315.05		315.03
36	Kaempferol-3-*O*-rutinoside (isomer)	7.85	264, 346	593.03	285.03			285.95
37	Kaempferol-3-*O*-rutinoside-7-*O*-rhamnoside	8.97		753.09	445.09	591.09	607.09	285.05
38	Myricetin-3-*O*-hexoside	9.62	355	479.11		317.11		317.11
39	Quercetin-3-*O*-(2-rhamnoside)-hexoside	9.87	366	609.05		447.05	463.05	301.05
40	Myricetin-3-*O*-rhamnoside	10.30	355	463.10			317,1	317.08
41	Quercetin-3-*O*-(2-rhamnoside)-hexoside	10.47	316	609.64		447.64	463.64	301.02
42	Myricetin-3-*O*-rhamnoside (isomer)	10.98	355	463.10			317.10	317.08
43	Kaempferol	11.43		285.95				285.95
44	Quercetin	11.71	256, 268sh, 352	301.04				301.02
**Flavan-3-ols**								
45	(+)-Catechin	3.41	278	289.06	245.14			
46	Procyanidin B2	3.73	280	577.03	289.04/245.14			
47	Procanidin C1	4.14	280	865.07	577.08/289.04/245.14			
48	(−)-Epicatechin	4.61	278	289.06	245.14			
49	Procyanidin dimer	5.56	280	577.04	289.04/245.14			

**Table 2:** Phenolic composition of caper flowers at different stage of development (mg/kg DW) ^‡^.

Cultivars	Stages of Development	Hydroxycinnamic Acid				Flavonols					F-3-ols	∑ Total polyphenols
		dpCA	dCA	dpQCA	dFQA	SA	dQ	dK	dISO	dM		
ORI.7	nonpareilles	46.4 ± 2.3	42.4 ± 2.4	18.5 ± 1.4	67.2 ± 2.1	8.3 ± 1.5	6254.9 ± 56	1511.0 ± 14	619.2 ± 12	1643.8 ± 23	513.2 ± 23	10724.9
	surfines	33.3 ± 2.1	30.1 ± 1.3	19.4 ± 1.6	90.1 ± 3.8	11.2 ± 1.1	5218.3 ± 57	2331.3 ± 15	618.3 ± 13	1607.4 ± 32	304.3 ± 19	10263.6
	capucines	19.3 ± 1.4	19.2 ± 2.1	20.8 ± 2.5	34.6 ± 2.6	9.6 ± 1.5	2245.6 ± 34	2133.5 ± 23	437.0 ± 21	1084.0 ± 11	670.7 ± 32	6674.3
	capotes	31.4 ± 2.1	16.6 ± 1.7	16.5 ± 1.7	24.3 ± 3.1	11.6 ± 1.6	2711.6 ± 21	1694.1 ± 14	355.2 ± 15	1046.1 ± 18	507.7 ± 26	6415.1
	fines	18.9 ± 1.6	20.2 ± 1.6	11.5 ± 1.4	25.4 ± 1.6	1.4 ± 0.4	1657.6 ± 32	815.0 ± 24	125.5 ± 17	361.6 ± 25	305.0 ± 29	3342.2
	gruesas	10.8 ± 0.9	22.9 ± 2.1	3.5 ± 2.6	13.7 ± 1.3	2.0 ± 0.2	1453.2 ± 19	979.1 ± 21	119.3 ± 10	317.7 ± 21	315.0 ± 32	3237.3
ORI.10	nonpareilles	32.9 ± 3.1	15.6 ± 2.7	5.4 ± 0.8	47.0 ± 1.9	5.0 ± 0.7	2375.6 ± 26	1885.1 ± 27	229.2 ± 9	760.6±25	208.0 ± 37	5564.5
	surfines	29.5 ± 2.7	14.8 ± 2.5	7.5 ± 1.1	51.1 ± 2.7	8.9 ± 1.1	2528.9 ± 21	1753.0 ± 31	230.8 ± 11	804.9 ± 31	344.0 ± 35	5773.4
	capucines	34.0 ± 4.1	17.2 ± 1.9	6.9 ± 0.5	14.4 ± 3.1	13.0 ± 0.9	2660.7 ± 22	1302.6 ± 16	141.0 ± 11	802.9 ± 27	366.8 ± 28	5359.4
	capotes	29.4 ± 2.5	15.3 ± 2.7	5.4 ± 0.9	21.5 ± 2.6	13.1 ± 1.1	2603.6 ± 32	1052.3 ± 14	225.7 ± 16	1018.7 ± 28	511.9 ± 26	5497.0
	fines	10.9 ± 1.8	15.6 ± 2.3	2.5 ± 0.3	14.7 ± 2.9	0.5 ± 0.2	2257.8 ± 15	503.4 ± 19	200.2 ± 14	430.1 ± 34	547.2 ± 21	3982.9
	gruesas	6.4 ± 0.4	11.5 ± 1.3	1.8 ± 0.4	14.4 ± 1.1	0.7 ± 0.1	2124.8 ± 19	532.5 ± 17	202.2 ± 18	322.6 ± 18	476.9 ± 22	3693.9
Stage of development	nonpareilles	a	a	a	a	b	a	c	a	a	a	a
	surfines	b	b	a	a	a	b	a	a	a	b	a
	capucines	c	bc	ab	b	ab	c	b	b	b	a	b
	capotes	d	c	b	c	ab	c	d	c	b	b	b
	fines	de	c	c	d	c	c	e	c	c	c	c
Cultivars	ORI.7	a	a	a	a	a	a	a	a	ab	a	a
	ORI.10	b	b	b	b	b	b	b	b	b	b	b

^‡^ Results are expressed as mean ± standard deviation of three determinations. F-3-ols—flavan-3-ols; dQ—derivatives of quercetin; dK—derivatives of kaempferol; dISO—derivatives of isorhamnetin; dM—derivaties of myricetin; dpCA—derivatives of *p*-coumaric acids; dCA—derivatives of caffeoylquinic acids; dpQCA—derivatives of *p*-coumaroylquinic acids; dFQA—derivatives of feruloylquinic acids; SA—sinapic acid; F-3-ols—sum of polymeric procyanidins; a, b, c letter were significantly different (p < 0.05) according to Duncan’s test;

**Table 3:** Antioxidant (ABTS, FRAP, ORAC), anti-diabetic (α-amylase and α-glucosidase), and cholinesterase’s (AChE and BuChE) inhibition of caper flowers at different stages of development ^‡^.

Cultivars	Stages of Development	Antioxidant Activity [mmol Trolox/100 g]			Anti-Diabetic Activity [IC_50_; mg/mL]		Cholinesterase’s Inhibition [% of Inhibition]	
		ABTS	FRAP	ORAC	α-amylase	α-glucosidase	AChE	BuChE
ORI.7	nonpareilles	6.92 ± 0.54	7.51 ± 0.11	27.66 ± 1.43	3.15 ± 0.11	2.98 ± 0.11	18.3 ± 0.1	31.0 ± 2.4
	surfines	6.89 ± 0.12	7.23 ± 0.47	25.52 ± 1.11	2.10 ± 0.05	2.47 ± 0.13	15.9 ± 0.3	20.1 ± 1.8
	capucines	6.53 ± 0.32	7.03 ± 0.72	22.97 ± 0.99	1.94 ± 0.21	2.32 ± 0.14	14.5 ± 0.7	28.4 ± 3.8
	capotes	6.05 ± 0.14	6.35 ± 0.32	22.25 ± 0.57	1.72 ± 0.13	2.20 ± 0.11	13.8 ± 1.7	20.5 ± 1.1
	fines	5.16 ± 0.21	6.76 ± 0.54	20.79 ± 1.17	1.30 ± 0.11	1.89 ± 0.11	12.1 ± 1.0	18.0 ± 1.2
	gruesas	3.56 ± 0.11	4.48 ± 0.38	16.77 ± 1.21	0.93 ± 0.07	1.52 ± 0.09	10.5 ± 0.9	11.4 ± 0.9
ORI.10	nonpareilles	6.82 ± 0.21	7.64 ± 0.51	19.27 ± 0.99	3.74 ± 0.99	4.46 ± 0.15	28.1 ± 2.0	33.8 ± 2.3
	surfines	5.83 ± 0.11	6.45 ± 0.58	18.55 ± 0.60	3.23 ± 0.21	3.68 ± 0.19	17.8 ± 1.3	25.2 ± 2.1
	capucines	5.45 ± 0.43	6.54 ± 0.32	16.45 ± 2.32	2.67 ± 0.15	2.38 ± 0.10	15.4 ± 0.9	23.4 ± 1.6
	capotes	2.43 ± 0.11	3.68 ± 0.24	15.29 ± 1.12	2.14 ± 0.21	2.34 ± 0.13	13.5 ± 1.3	20.4 ± 1.4
	fines	2.01 ± 0.09	2.83 ± 0.44	13.59 ± 1.43	1.71 ± 0.32	2.02 ± 0.06	13.4 ± 1.4	19.2 ± 1.8
	gruesas	0.54 ± 0.04	1.69 ± 0.37	10.09 ± 2.01	1.45 ± 0.15	1.97 ± 0.89	10.4 ± 1.2	8.6 ± 1.2
Stage of development	nonpareilles	a	a	a	a	a	a	a
	surfines	a	a	ab	a	b	b	ab
	capucines	a	ab	ab	ab	c	bc	ab
	capotes	b	b	b	b	c	c	b
	fines	b	b	b	bc	d	c	b
Cultivars	ORI.7	a	a	a	a	a	a	a
	ORI.10	b	b	b	b	b	b	b

^‡^ The results are shown as mean ± standard deviation of three assays performed in triplicate. AChE—acetylcholinesterase; BuChE—butyrylcholinesterase. (2,2′-azinobis-(3-ethylbenzothiazoline-6-sulfonic acid)] radical cation (ABTS•+), ferric-reducing antioxidant power (FRAP), and Oxygen Radical Absorbance Capacity (ORAC), a, b, c letter were significantly different (p < 0.05) according to Duncan’s test;

**Table 4:** Pearson’s correlation coefficients between in vitro biological activity methods and polyphenols content in caper flowers.

Accession	ABTS	FRAP	ORAC	AChE	BuChE	α-amylase	α-glucosidase
∑ Flavonols	0.666	0.598	0.805	0.393	0.547	0.497	0.357
∑ Quercetin derivatives	0.474	0.419	0.684	0.285	0.408	−0.301	−0.220
∑ Kaempferol derivatives	0.855	0.791	0.737	0.550	0.641	0.025	−0.438
∑ Isorhamnetin derivatives	0.556	0.466	0.786	0.226	0.423	−0.422	−0.181
∑ Myricetin derivatives	0.652	0.585	0.790	0.348	0.571	−0.217	−0.403
∑ Aglycone of flavonols	0.460	0.425	0.635	−0.170	0.278	−0.140	−0.257
∑ Mono-*O*-glycosides of flavonols	0.546	0.513	0.677	0.490	0.601	0.638	0.464
∑ Di-*O*-glycosides of flavonols	0.480	0.425	0.689	0.291	0.412	0.434	0.258
∑ Tri-*O*-glycosides of flavonols	0.546	0.468	0.806	0.183	0.379	0.224	0.105
∑ Tetra-*O*-glycosides of flavonols	0.479	0.420	0.784	0.027	0.251	0.092	−0.023
∑ Phenolic acid	0.571	0.582	0.287	0.866	0.765	0.925	0.871
∑ *p*-CA derivatives	0.737	0.730	0.694	0.605	0.732	0.085	−0.607
∑ CA derivatives	0.506	0.487	0.772	0.120	0.309	−0.458	−0.398
∑ *p*-QCA derivatives	0.747	0.699	0.906	0.102	0.445	−0.286	−0.371
∑ FQA derivatives	0.676	0.628	0.753	0.498	0.496	−0.268	−0.233
SA	0.496	0.460	0.428	0.204	0.434	0.270	−0.516
∑ flavan-3-ols	−0.239	−0.307	−0.009	−0.403	0.005	−0.268	0.153

pCA—derivatives of *p*-coumaric acids; CA—derivatives of caffeoylquinic acids; *p*-QCA—derivatives of *p*-coumaroylquinic acids; FQA—derivatives of feruloylquinic acids; SA—sinapic acid; correlation is significant at the 0.05 level.
